# Overexpressed histone acetyltransferase 1 regulates cancer immunity by increasing programmed death-ligand 1 expression in pancreatic cancer

**DOI:** 10.1186/s13046-019-1044-z

**Published:** 2019-02-01

**Authors:** Ping Fan, Jingyuan Zhao, Zibo Meng, Heyu Wu, Bo Wang, Heshui Wu, Xin Jin

**Affiliations:** 1 Department of Pancreatic Surgery, Union Hospital, Tongji Medical College, Huazhong University of Science and Technology, Wuhan, 430022 China; 2 Operating Room, Union Hospital, Tongji Medical College, Huazhong University of Science and Technology, Wuhan, 430022 China

**Keywords:** Histone acetyltransferase 1, Pancreatic ductal adenocarcinoma, PD-L1

## Abstract

**Background:**

Pancreatic ductal adenocarcinoma is one of the leading causes of cancer-related death worldwide. Immune checkpoint blockade therapy, including anti-PD-1 and anti-PD-L1, is a new therapeutic strategy for cancer treatment but the monotherapy with PD-L1 inhibitors for pancreatic cancer is almost ineffective for pancreatic cancer. Thus, exploring the regulatory mechanism of PD-L1 in cancer cells, especially in pancreatic cancer cells, is one of the key strategies to improving cancer patient response to PD-L1 blockade therapy. Histone acetyltransferase 1(HAT1) is a classic type B histone acetyltransferase and the biological role of HAT1 in pancreatic cancer is unclear.

**Methods:**

The clinical relevance of HAT1 was examined by the GEPIA web tool, Western blotting and immunohistochemistry of pancreatic cancer tissue microarray slides. Tumor cell motility was investigated by MTS assay, colony formation assay and xenografts. The relationship between HAT1 and PD-L1 was examined by Western blot analysis, RT-qPCR and immunohistochemistry.

**Results:**

HAT1 was upregulated in PDAC and associated with poor prognosis in PDAC patients. The knockdown of HAT1 decreased the proliferation of pancreatic cancer cells in vivo and in vitro. Strikingly, we showed that HAT1 transcriptionally regulated PD-L1, and this process was mainly mediated by BRD4 in pancreatic cancer. The knockdown of HAT1 improved the efficacy of immune checkpoint blockade by decreasing the PD-L1.

**Conclusions:**

The recognition of HAT1 in regulating tumor cell proliferation and cancer immunity indicated that HAT1 might be employed as a new diagnostic and prognostic marker and a predictive marker for pancreatic cancer therapy, especially in immune checkpoint blockade therapy. Targeting HAT1 highlights a novel therapeutic approach to overcome immune evasion by tumor cells.

**Electronic supplementary material:**

The online version of this article (10.1186/s13046-019-1044-z) contains supplementary material, which is available to authorized users.

## Background

Pancreatic ductal adenocarcinoma (PDAC) is one of the leading causes of cancer-related death worldwide [1]. Resistance to chemotherapy and radiotherapy results in the poor prognosis of PDAC [2]. Immunotherapy is a new therapeutic strategy for cancer treatment and has made a profound progress in prolonging the survival time patients with of various types of tumors [3]. However, the immunotherapy is almost ineffective for pancreatic cancer [4]. Therefore, exploring the underlying mechanisms is urgently needed to overcome the resistance to immunotherapy in pancreatic cancer.

Tumors evade immune surveillance by the aberrant activation of inhibitory pathways that regulate the function of T lymphocytes, known as immune checkpoints [5]. Programmed death-ligand 1 (PD-L1, B7-H1) is a member of the B7 family of cell surface ligands on cancer cells surfaces, which binds the programmed death-1 protein (PD-1) receptor to induce T cell apoptosis and inhibit cytotoxic T-cell activation within cancer tissues [6–9]. Given that the blockade of the PD-1/PD-L1 interaction can reactivate T-cell responses, a few anti-PD-1 and anti-PD-L1 antibodies have been approved for the treatment of human cancers in the clinic [10]. However, monotherapy with PD-L1 inhibitors for pancreatic cancer has resulted in disappointing outcomes in clinical trials [11]. A growing body of evidence suggests that the expression level of PD-L1 in cancer cells is highly associated with the response to immune checkpoint therapies [12]. Thus, exploring the regulatory mechanism of PD-L1 in cancer cells, especially in pancreatic cancer cells, is one of the key strategies to improve cancer patient response to PD-L1 blockade therapy.

Histone acetyltransferase 1 (HAT1) is a classic type B histone acetyltransferase, and it can only acetylate newly synthesized histone H4 and not nucleosomal histone [13].HAT1 was the first histone acetyltransferase identified and is one of the most poorly understood members of this family [13]. HAT1 is overexpressed in multiple types of solid tumors, including esophageal [14], lung cancer [15] and liver cancer [16], and acts as an oncoprotein to promote tumorigenesis. It has been reported that HAT1 functions as a transcription factor to regulate the expression of various genes, such as Bcl2L12 [17] and Fas [15], and modulates cancer cell proliferation [16], apoptosis [15] and metabolism [16].

To date, the biological effect and clinical relevance of HAT1 in pancreatic cancer is poorly understood. In this study, we sought to determine the specific role of HAT1 in pancreatic cancer. First of all, we demonstrated that HAT1 was overexpressed in pancreatic cancer and linked with poor prognosis in PDAC patients. Then, our data showed that HAT1 acted as a tumor growth promoting protein in pancreatic cancer cells. Strikingly, HAT1 was involved in the cancer immunity response by regulating the PD-L1 expression, and this process was mainly mediated by BRD4. Taken together, our results demonstrate that aberrant expression of HAT1 promotes tumorigenesis by modulating the cancer cell growth and the immune response in pancreatic cancer.

## Materials and methods

### Cell culture

All pancreatic cancer cell lines including PANC-1, BxPC-3 and MIA PaCa-2 were purchased from the Chinese Academy of Science Cell Bank, and the Panc 02 cells were obtained from Tong Pai Technology (Shanghai, China). These cell lines were cultured in Dulbecco’s Modified Eagle Medium (DMEM) medium (Invitrogen, USA) supplemented with 10% fetal bovine serum (FBS) (HyClone, USA). All cell lines were routinely maintained at 37 °C in a 5% CO_2_ incubator.

### Plasmids, antibodies and chemicals

Mammalian expression vectors for Flag-HAT1 recombinant proteins were generated using the pcDNA3.1 backbone vector. The HAT1 antibody (ab194296) was purchased from Abcam (working dilution 1:2000); beta-tubulin (2128S) was from Cell Signaling Technology - (working dilution 1:5000); BRD4 (ab128874) was from Abcam (working dilution 1:1000); PD-L1 (13684S) was from Cell Signaling Technology (working dilution 1:1000); and H4K5ac (ab17343) was from Abcam (working dilution 1:1000). Ascorbate was purchased from Sigma-Aldrich (Shanghai, China).

### Western blot of cells and tissue specimens

The ethics of using human tissue (12 pairs of matched pancreatic cancer/adjacent noncancerous tissues) was approved by the local ethics committee (Tongji Medical College, China), and written informed consent was obtained from patients prior to surgery exactly as described previously [18]. The cells or the tissue specimens were lysed with lysis buffer (Beyotime, China) containing 1% protease and phosphatase inhibitors. The protein concentration was determined with a protein assay kit (Pierce Biotechnology, USA). Equal amounts of protein for each sample were separated using SDS-PAGE gels and transferred onto PVDF membranes (Pierce Biotechnology, USA). The membranes were subsequently blocked in 5% not-fat milk for 1 h at room temperature, followed by incubation with primary antibody overnight at 4 °C. The membranes were then washed with 1x TBST and incubated with a secondary antibody for 1 h. Finally, the membranes were treated with ECL detection reagents and exposed to X-ray films.

### Real-time RT-PCR

Total RNA was extracted from the cells using Trizol reagent (Thermo Fisher Scientific, USA). First strand cDNA was synthesized from 2 μg RNA using a cDNA Reverse Transcription kit (PrimeScript™ RT reagent Kit, Code No. RR037A), and real-time PCR analysis was carried out with a PCR kit (TB Green™ Fast qPCR Mix, Code No. RR430A) according to the manufacturer’s protocols. The two kits were purchased from Takara Bio Inc. (Shigo, Japan). All the values were normalized to actin, and the 2-ΔCt method was used to quantify the fold change. The primers used for RT-qPCR are provided in Additional file 1: Table S1.

### Chromatin immunoprecipitation (ChIP) and ChIP-qPCR

ChIP was performed following the manufacturer’s instructions for the Chromatin Extraction Kit (Abcam, ab117152, USA) and ChIP Kit Magnetic - One Step (Abcam, ab156907, USA) [19]. BRD4 (Cell Signaling Technology, 13,440, dilution 1:50) was used for the ChIP assay. The purified DNA was analyzed by real-time PCR with a PCR kit (Takara Bio Inc., Japan) according to the manufacturer’s protocols [20]. The primers for ChIP-qPCR are provided in Additional file 1: Table S2.

### Tissue microarray and immunohistochemistry (IHC)

The tissue microarray slides were purchased from Outdo Biobank (Shanghai, China) (HPan-Ade060CD-01). The tissue microarray specimens were immunostained with PD-L1 (Cell Signaling Technology, 13,684, dilution 1: 1000) and HAT1 antibodies (Abcam, ab194296, dilution 1:3000) as described previously. Staining intensity was scored in a blinded fashion: 1 = weak staining at 100× magnification but little or no staining at 40× magnification; 2 = medium staining at 40× magnification; 3 = strong staining at 40× magnification [21]. The degree of immunostaining was reviewed and scored by two independent pathologists who were blinded to the clinical details. The scores were determined by the percentage of positive cells multiplied by the staining intensity.

### RNA interference

The lentivirus-based control and gene-specific shRNAs were purchased from Sigma-Aldrich. Lipofectamine 2000 was used to transfect 293 T cells with shRNA plasmids and viral packaging plasmids (pVSV-G and pEXQV). Twenty-four hours after transfection, the medium was replaced with fresh DMEM, containing 10% FBS and 1 mM of sodium pyruvate. Next, 48 h post transfection, the virus culture medium was collected and added to the PANC-1, MIA PaCa-2 and BxPC-3 cells supplemented with 12 μg/ml of polybrene. Twenty-four hours after infection, the infected cells were selected with 10 μg /ml of puromycin. The shRNA sequence information is provided in Additional file 1: Table S3.

The pTsin lentiviral expression vector was used to generate lentiviral plasmids for pTsin-Flag-HAT1. Lipofectamine 2000 was used to transfect 293 T cells with the pTsin expression plasmid and viral packaging plasmids (pHR’ CMVδ 9.8 and pVSV-G). Twenty-four hours after transfection, the medium was replaced with fresh DMEM, containing 10% FBS and 1 mM of sodium pyruvate. Next, 48 h post transfection, the virus culture medium was collected and added to PANC-1 cells supplemented with 12 μg/ml of polybrene. Twenty-four hours after infection, the infected cells were selected with 10 μg/ml of puromycin.

### Cell proliferation assay

Cell viability was evaluated using the MTS (3-(4,5-dimethylthiazol-2-yl)-5-(3-carboxymethoxyphenyl)-2-(4-sulfophenyl)-2H-tetrazolium, inner salt) assay according to the manufacturer’s instructions (Abcam, USA). Briefly, the pancreatic cancer cells (1 × 10^3^ cells) were seeded in 96-well plates with 100 μl of culture medium. The cells were treated with serial concentrations of small molecular inhibitors. After 72 h, 20 μl of MTS reagent (Abcam, USA) was added to each well of the cells and incubated for 1 h at 37 °C in standard culture conditions. The absorbance was measured in a microplate reader at 490 nm.

### Generation of PDAC xenografts in nude mice

The BALB/c-nu mice (4–5 weeks of age, 18–20 g) were purchased from Vitalriver (Beijing, China) and randomly divided into two groups (*n* = 7/group) for the subcutaneous inoculation with 5 × 10^6^ of PANC-1 cells infected with shControl or shHAT1 lentivirus in the left dorsal flank of the mice. The tumors were examined every other day for 21 days; the length and width measurements were obtained with calipers to calculate the tumor volumes by using the eq. (L x W^2^)/2. On day 21, the animals were euthanized, and the tumors were excised and weighed. All the animal experimental procedures were approved by the Ethics Committee of Tongji Medical College, Huazhong University of Science and Technology.

### Survival analysis and correlation analysis using the GEPIA web tool

The online database Gene Expression Profiling Interactive Analysis (GEPIA, http://gepia.cancerpku.cn/index.html.) [22] was used to analyze the RNA sequencing expression data related to our project based on The Cancer Genome Atlas (TCGA) and the Genotype-Tissue Expression (GTEx) projects. GEPIA performs survival analyses based on gene expression levels and uses a log-rank test for hypothesis evaluation. GEPIA performs a pairwise gene correlation analysis for any given set of TCGA and/or GTEx expression data using Pearson correlation statistics.

### Generation and treatment of Panc 02 xenografts in mice

Six-week-old C57BL/6 mice were purchased from Charles River Laboratories (Wuhan, China). All the animal experimental procedures were approved by the Ethics Committee of Tongji Medical College, Huazhong University of Science and Technology. Panc 02 cells (5 × 10^6^ in 100 μl 1 × PBS) infected with shControl or shHAT1 lentivirus were injected s.c. into the right flank of mice. The volume of xenografts was measured every other day and calculated using the formula LxW^2^x0.5. After the xenografts reached a size of approximately 50 mm^3^, mice carrying similar types of tumors were randomized into different groups and treated with anti-PD-1 (BioXcell, Clone RMP1–14)/IgG (BioXcell, Clone 2A3) (200 μg, i.p., given at days 0, 3, 6); or anti-PD-L1 (BioXcell, Clone 10F.9G2)/IgG (BioXcell, Clone MPC-11) (200 μg, i.p., given at days 0, 3, 6). Mice were euthanized and tumors were collected from all animals once the tumors reached a volume of 200 mm^3^.

### Flow cytometry analysis

The PANC-1, MIA PaCa-2 and BxPC-3 cells infected with control or HAT1-specific shRNAs were harvested and washed with PBS. Cells were fixed in 4% paraformaldehyde for 15 min. After washing with PBS, cells were incubated with ice-cold 100% methanol for 30 min on ice. Cells were washed with PBS and incubated with PD-L1 antibody (Biolegend, APC anti-human CD274, clone 29E.2A3) or isotype IgG (Biolegend,APC anti-human IgG Fc Antibody, clone HP6017) for 15 min at room temperature. After washing three times with PBS, the cells were resuspended in PBS and analyzed by flow cytometry.

For flow cytometry analysis of the mouse tissue samples, the tumors were cut into small pieces and digested with 2 mg/ml collagenase (Sigma, USA) in DMEM for 1 h at 37 °C. Cells were filtered through a 70 μm nylon strainer and resuspended in red blood cell lysis buffer (Biolegend) for 3 min at room temperature. The cells were then suspended in PBS with 2% BSA and costained with the following antibodies: CD45 (Biolegend, 103,112, APC conjugated); CD4 (Biolegend, 100,510, FITC conjugated); CD8 (Biolegend, 100,708, PE conjugated); CD11b (Biolegend,101,212, APC conjugated); and Gr1 ((Biolegend, 108,406, FITC conjugated)). After incubation with antibody for 15 min, the cells were washed with PBS and analyzed by flow cytometry.

### Statistical analysis

Statistical analyses were performed with one-sided or two-sided paired Student’s t-test for single comparison and one-way ANOVA with a post hoc test for multiple comparisons. A *P* value < 0.05 was considered statistically significant. All the values are expressed as the mean ± SD.

## Results

### HAT1 is up-regulated in PDAC and associated with poor prognosis in PDAC patients

**Fig. 1:**
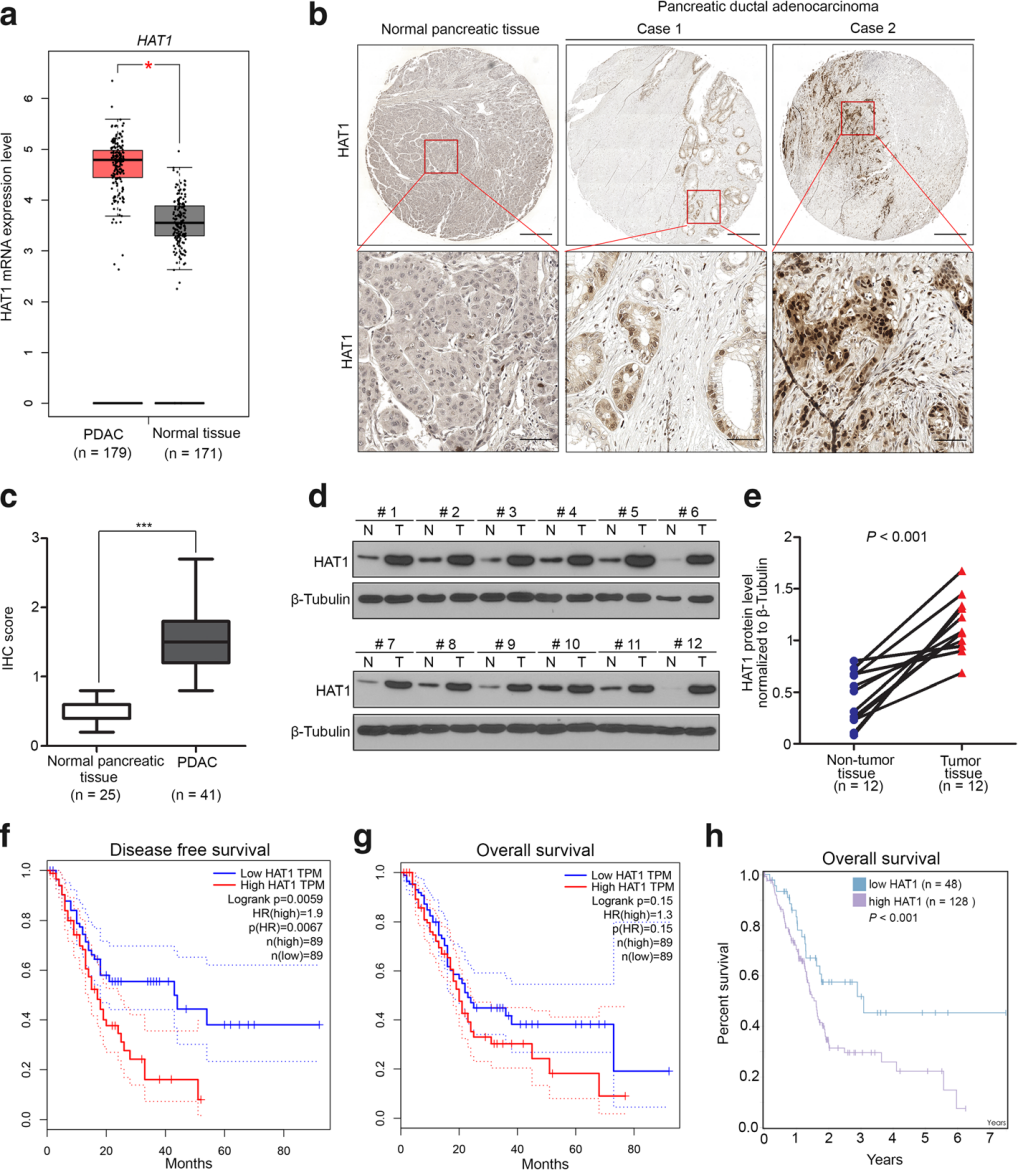
HAT1 is upregulated in PDAC and associated with poor prognosis in PDAC patients. **a**, The GEPIA database revealed that HAT1 expression was significantly upregulated in pancreatic cancer tissues. The boxplot analysis show log2 (TPM + 1) on a log-scale. **b**, Images of the IHC analyses of HAT1 using TMA (*n* = 25 normal pancreatic specimens, *n* = 41 PDAC) tissue sections. Scale bars are shown as indicated. **c**, Box plots of HAT1 expression as determined by the IHC score using TMA (n = 25 normal pancreatic specimens, n = 41 PDAC) tissue sections. ***, *P* < 0.001. **d** and **e**, for **d**, Expression of HAT1 as determined by Western blot analysis, in eight paired primary pancreatic cancer tissues (T) and the matched adjacent nontumor tissues (N) from the same patient. β -Tubulin served as a loading control; for **e**, HAT1 proteins were quantified by ImageJ software. The *P* values are also shown. **f** and **g**, The disease-free and (f) overall survival (g) of the patients with PDAC were computed with the GEPIA web tool. **h**, The overall survival of the patients with PDAC was computed with the Human Protein Atlas

To investigate the expression level of HAT1 in pancreatic cancer, we first analyzed *HAT1* mRNA levels in pancreatic cancer and nontumor pancreatic tissues by using the GEPIA web tool [22]. We found that the mRNA levels of *HAT1* in pancreatic cancer tissues were higher than in nontumor pancreatic tissues (Fig. 1a). Then, we sought to determine the HAT1 protein levels in human PDAC specimens via using the TMA (tissue microarray) approach. We examined the protein level of the HAT1 by immunohistochemistry (IHC) in PDAC specimens obtained from a cohort of patients (*n* = 25 normal pancreatic specimens, *n* = 41 PDAC TMA specimens). The IHC staining score was evaluated by measuring both the percentage of cells that stained positive for the marker and the staining intensity [20]. We showed that HAT1 was significantly overexpressed in PDAC specimens compared with normal pancreatic tissues (Fig. 1b and c). Similarly, we examined the protein level in PDAC and paired adjacent nontumor pancreatic tissues in our hospital via Western blot analysis and demonstrated that HAT1 was upregulated in PDAC compared to adjacent non-tumor pancreatic tissues (Fig. 1d and e). To further identify the clinical relevance of HAT1 in pancreatic cancer, the survival rate of PDAC patients associated with HAT1 expression was determined through GEPIA web tool and The Human Protein Atlas. Our data indicated that the high expression of HAT1 was closely correlated with poor prognosis in PDAC patients (Fig. 1f-g). Together, these data suggest that HAT1 is upregulated in PDAC and associated with poor prognosis in PDAC patients.

### HAT1 promotes cell proliferation in pancreatic cancer in vivo and in vitro

**Fig. 2:**
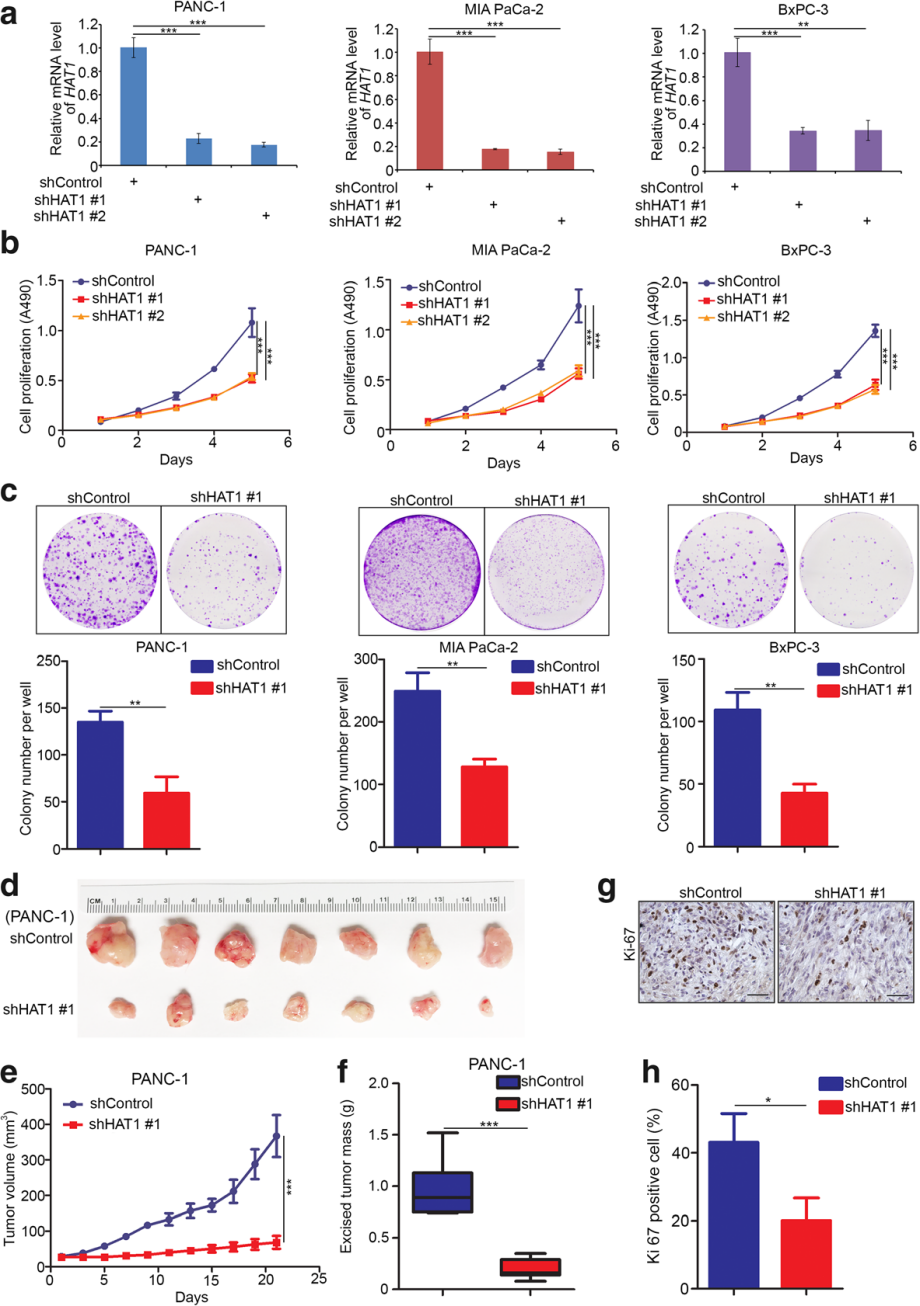
HAT1 promotes cell proliferation in pancreatic cancer in vivo and in vitro. **a-c**, PANC-1, MIA PaCa-2 and BxPC-3 cells were infected with lentivirus vectors expressing control or HAT1-specific shRNAs. Forty-eight hours postinfection, the cells were harvested for RT-qPCR analysis (**a**), MTS assay (**b**) and colony formation assay (**c**). The data shown are the mean values ± SD from three replicates. **, *P* < 0.01; ***, *P* < 0.001. **d-f**, PANC-1 cells were infected with control or HAT1-specific shRNAs. Then, 72 h postinfection, the cells were injected subcutaneously into the right dorsal flank of nude mice. After 24 days, the tumors were harvested, photographed (d) and measured (e and f). The data are presented as the mean ± SD (*n* = 7). ***, *P* < 0.001. **g** and **h**, IHC analysis of the Ki-67 expression in xenografts was performed, and the staining was quantified. All data shown are mean ± SD (error bar) from five replicates. *, *P* < 0.05

Given that HAT1 functioned as a negative prognostic biomarker in PDAC, we wanted to explore the specific role of HAT1 in pancreatic cancer. First, we knocked down HAT1 with a specific lentiviral short hairpin RNA in PANC-1, MIA PaCa-2 and BxPC-3 cells (Fig. 2a). The MTS assay and colony formation assay indicated that the knock down of HAT1 significantly impeded the cell growth of the PANC-1, MIA PaCa-2 and BxPC-3 cells (Fig. 2b and c). On the other hand, we also found that the overexpression of HAT1 promoted the proliferation of PANC-1 and BxPC-3 cells (Additional file 1: Figure S1a and b). The above data were consistent with the data reported for liver, nasopharyngeal and lung cancer cells [15–17]. Moreover, to investigate the role of HAT1 in the tumor growth of PDAC in vivo, PANC-1 cells infected with control or HAT1-specific shRNAs were injected subcutaneously into the right flank of nude mice for the xenograft assay. We found that the knockdown of HAT1 blocked the growth of PANC-1 xenografts in mice (Fig. 2d-f). Then, xenografts were subjected to IHC analysis for Ki-67 expression, the most commonly used indicator to evaluate cell proliferation (Fig. 2g). We found that the knockdown of HAT1 resulted in a decrease in Ki-67 staining compared with the control group (Fig. 2h). Furthermore, the PANC-1 cells infected with pTsin-EV or pTsin-Flag-HAT1 used to establish the control or HAT1-overexpressing pancreatic cancer stable cell lines, respectively, were injected subcutaneously into the right flank of nude mice for the xenograft assay. Our data demonstrated that overexpressed HAT1 promoted pancreatic cancer growth in vivo (Additional file 1: Figure S1c-e). Taken together, our findings indicate that HAT1 acts as a growth promoting protein in pancreatic cancer.

### HAT1 transcriptionally increases PD-L1 expression in pancreatic cancer cells

**Fig. 3:**
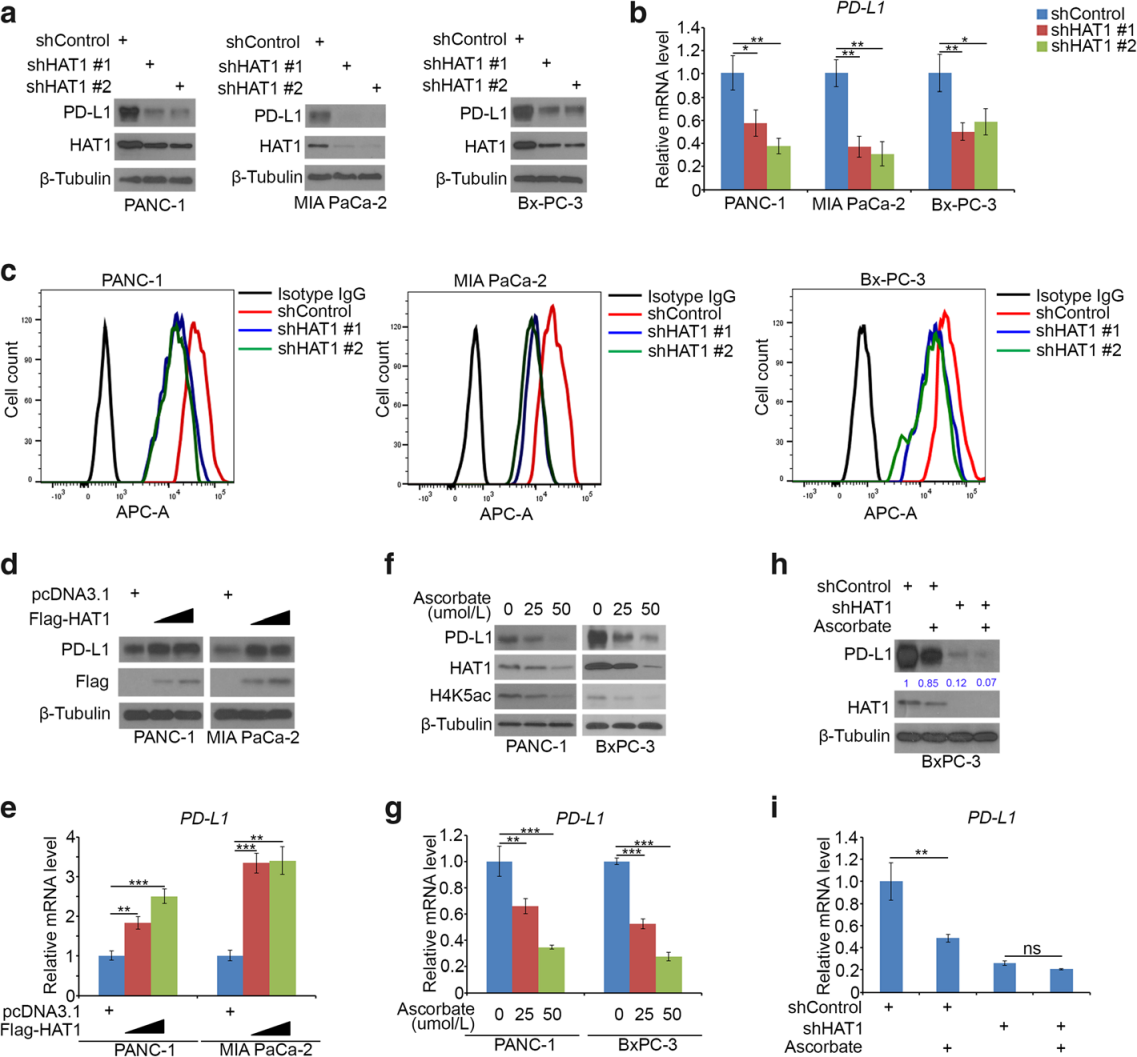
HAT1 transcriptionally increases PD-L1 expression in pancreatic cancer cells. **a-c**, PANC-1, MIA PaCa-2 and BxPC-3 cells were infected with lentivirus vectors expressing control or HAT1-specific shRNAs. Forty-eight hours postinfection, the cells were harvested for Western blotting (a), RT-qPCR analysis (b) and FACS analysis (c). The data shown are the mean values ± SD from three replicates. *, *P* < 0.05; **, *P* < 0.01. **d** and **e**, PANC-1 and MIA PaCa-2 cells were transfected with pcDNA3.1, 1 μg Flag-HAT1 or 4μg Flag-HAT1 plasmids. Then, 24 h posttransfection, cells were harvested for Western blotting (d) and RT-qPCR analysis (e). The data shown are the mean values ± SD from three replicates. **, *P* < 0.01; ***, *P* < 0.001. **f** and **g**, PANC-1 and BxPC-3 cells were treated with serial concentrations of ascorbate for 24 h and cells were harvested for Western blotting (f) and RT-qPCR analysis (g). The data shown are the mean values ± SD from three replicates. **, *P* < 0.01; ***, *P* < 0.001.**h** and **i**, BxPC-3 cells were infected with the indicated constructs. After 48 h, cells were treated with or without ascorbate for another 24 h and the cells were harvested for Western blotting (h) and RT-qPCR analysis (i). The data shown are the mean values ± SD from three replicates. ns, not significant; **, *P* < 0.01

Since HAT1 was upregulated in PDAC and promoted cell proliferation in pancreatic cancer cells, the more biological role of HAT1 needs to be explored. It has been documented that PD-L1 was inversely correlated with prognosis in pancreatic cancer [23]. Given the poorly understood nature of its regulation [24], we sought to determine whether HAT1 is involved in PD-L1 regulation. Strikingly, the knockdown of HAT1 decreased the protein and mRNA expression of PD-L1 in the PANC-1, MIA PaCa-2 and BxPC-3 cells (Fig. 3a-c). Conversely, using a gain of function approach, we demonstrated that the ectopic expression of HAT1 led to the up-regulation of PD-L1 expression in both PANC-1 and MIA PaCa-2 cells (Fig. 3d and e). A recent study reported that HAT1 was downregulated by ascorbate through the TET-mediated DNA hydroxymethylation pathway [25]. Similarly, the ascorbate treatment decreased the protein and mRNA levels of both PD-L1 and HAT1 in the PANC-1 and BxPC-3 cells (Fig. 3f and g). Then, we sought to examine whether the effect of ascorbate on PD-L1 expression is mediated by HAT1 or not. The ascorbate treatment decreased PD-L1 expression, and this effect was diminished after HAT1 knockdown in BxPC-3 cells (Fig. 3h and i), which indicated that HAT1 played a key role in modulating the ascorbate-induced PD-L1 down-regulation of PD-L1. Taken together, our findings indicated that HAT1 upregulated PD-L1 expression at the transcriptional level.

### PD-L1 is positively correlated with HAT1 in PDAC patient specimens

**Fig. 4:**
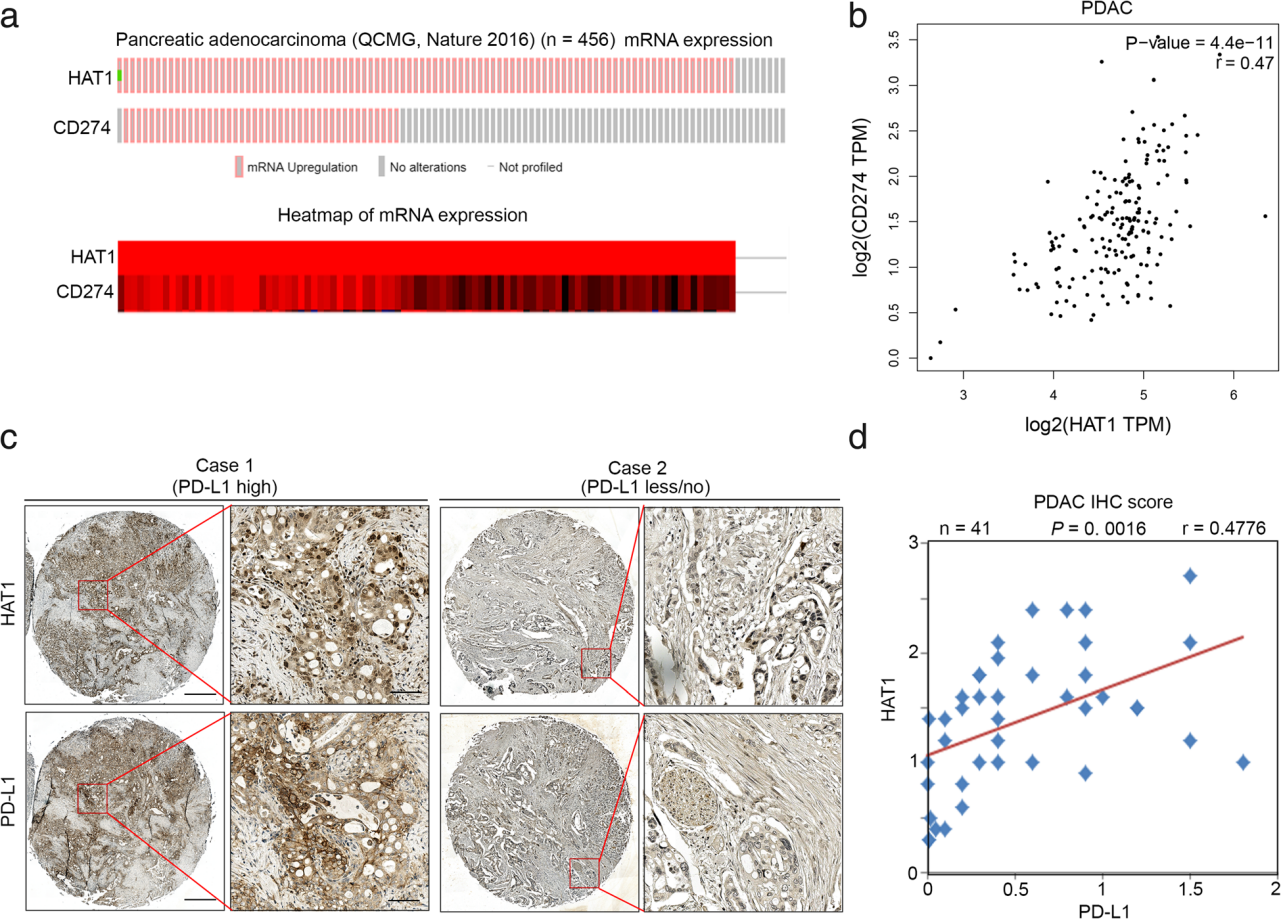
PD-L1 is positively correlated with HAT1 in PDAC patient specimens. **a**, The mRNA levels of PD-L1 (CD274) and HAT1 as well as the corresponding heatmap of pancreatic cancer dataset reported by the QCMG group (*n* = 456) [26]. **b**, The GEPIA web tool was used to determine the correlation between the mRNA expression levels of PD-L1 and HAT1 in human pancreatic cancer samples. **c**, Images of the IHC analysis of PD-L1 and HAT1 using TMA (*n* = 41 PDAC) tissue sections. Scale bars are shown as indicated. **d**, Correlation analysis of the staining index for the expression of the HAT1 and PD-L1 proteins in PDAC patient specimens (n = 41). Pearson product-moment correlation coefficients and the *P* values are also shown

To further investigate the relationship between PD-L1 and HAT1, we analyzed the mRNA levels of *PD-L1 (CD274)* and *HAT1* in a subset of pancreatic cancer patients (Fig. 4a) [26]. Intriguingly, we found that the overexpression of *PD-L1 (CD274)* was accompanied by the upregulation of *HAT1* (*p* < 0.05) (Fig. 4a). Then, we analyzed the correlation of the mRNA level between PD-L1 and HAT1 using the GEPIA web tool. Our results indicated that PD-L1 mRNA is positively correlated with HAT1 mRNA in pancreatic cancer patient specimens (Pearson’s product-moment correlation coefficient r = 0.47, *p* = 4.4e-11) (Fig. 4b). To further determine the correlation between HAT1 and PD-L1 in pancreatic cancer specimens, we examined the expression of these two proteins by performing immunohistochemistry (IHC) on a tissue microarray (TMA) containing a cohort of pancreatic cancer samples (*n* = 41). The IHC staining index (SI) was calculated by multiplying the percentage of positively stained cells and the staining intensity. Representative images of high and low/no staining of HAT1 and PD-L1 are shown in Fig. 4c. The PD-L1 expression was positively correlated with the HAT1 level (Pearson’s product-moment correlation coefficient r = 0.4776, *p* = 0.0016) (Fig. 4d), which was consistent with the mRNA level correlation reported above. Together, our results suggest that PD-L1 is positively correlated with HAT1 in PDAC patient specimens.

### Knockdown of HAT1 improves the efficacy of immune checkpoint blockade by decreasing the PD-L1 expression in vivo

**Fig. 5:**
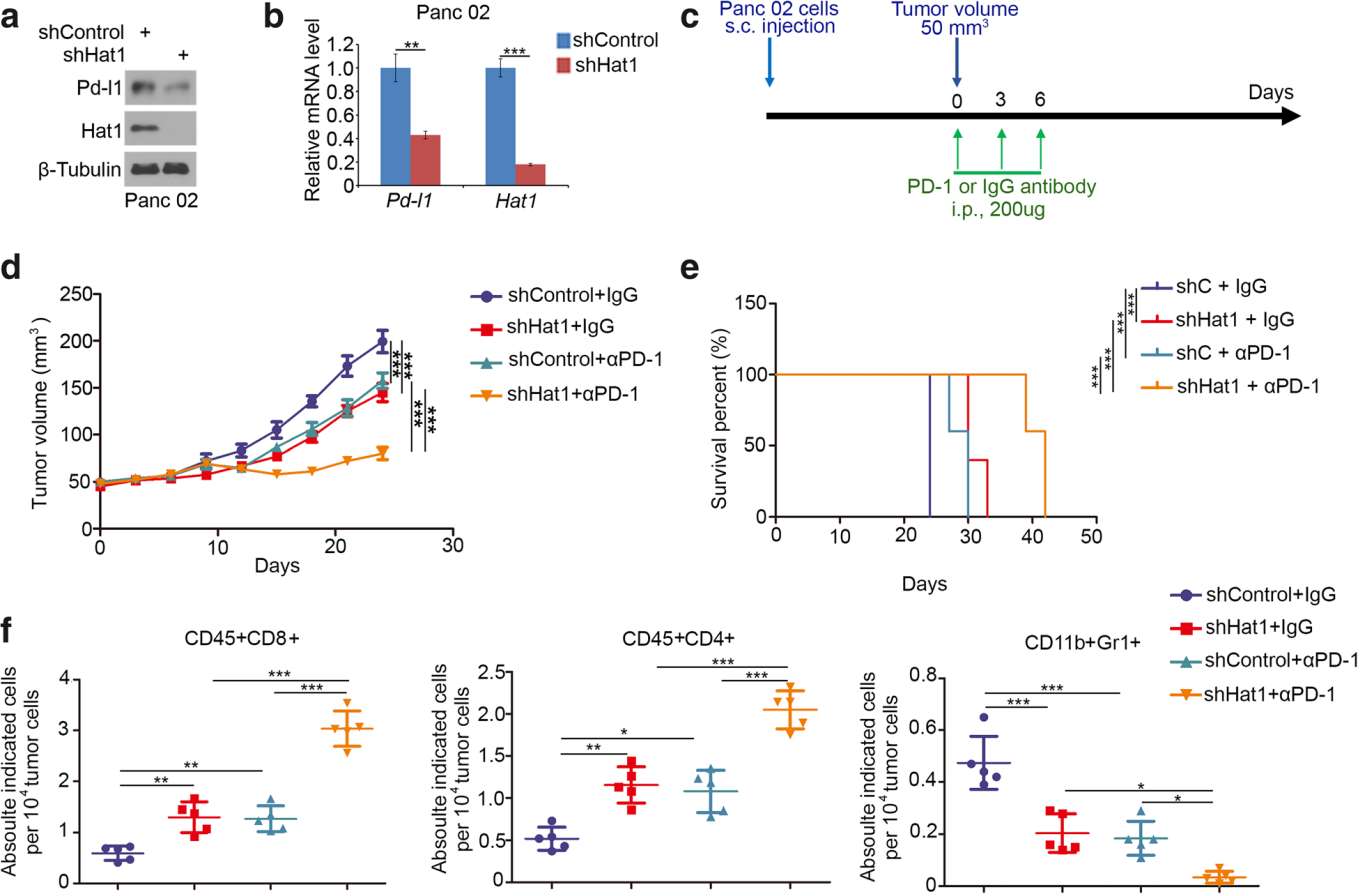
Knockdown of HAT1 improves the efficacy of immune checkpoint blockade by decreasing the PD-L1 expression in vivo. **a** and **b**, Panc 02 cells were infected with lentivirus vectors expressing control or Hat1-specific shRNAs. Forty-eight hours postinfection, the cells were harvested for Western blotting (**a**) and RT-qPCR analysis (**b**). The data shown are the mean values ± SD from three replicates. **, *P* < 0.01; ***, *P* < 0.001. **c**, Schematic diagram depicting the treatment plan for mice bearing subcutaneous Panc 02 tumors. **d**, Panc 02 cells were infected with lentivirus vectors expressing control or Hat1-specific shRNAs. Seventy-two hours after selection with puromycin, 5 × 10^6^ cells were injected subcutaneously into C57BL/6 mice. Mice (*n* = 5/group) were treated with anti-PD-1 (200 μg) or nonspecific IgG for 42 days. The growth curves of tumors with the different treatments are shown in (**d**). **e**, Kaplan-Meier survival curves for each treatment group demonstrate the improved efficacy of combining PD-1 mAb with HAT1 knockdown. ***P < 0.001. (Gehan-Breslow-Wilcoxo test). **f**, At the end of the treatment, the numbers of infiltrated CD45^+^CD8^+^ T cells, CD45^+^CD4^+^ T cells, and CD11b^+^Gr1^+^ myeloid cells that infiltrated tumors following the different treatments were analyzed by FACS. All data are shown as the mean values ± SD. ns, not significant, * *P* < 0.05, ** *P* < 0.01, *** *P* < 0.001

As described above, HAT1 regulated PD-L1 expression in human pancreatic cancer cells. We sought to examine this phenomenon in vivo. First, we knocked down of Hat1 in the murine pancreatic cancer cell line Panc 02 and showed that reducing the Hat1 resulted in decreasing the Pd-l1 (Fig. 5a and b). These results suggest that the knockdown of Hat1 may enhance the immune checkpoint blockade efficacy in pancreatic cancer therapy. To test this hypothesis, Panc 02 cells infected with shControl or shHat1 lentivirus were injected subcutaneously into immune-proficient mice. Panc 02 tumor-bearing mice were sham-treated or treated with anti-PD-1 antibody or nonspecific control IgG as indicated in Fig. 5c. Consistent with the previous results in human pancreatic cancer cells (Fig. 2), the knockdown of Hat1 slowed down the cells proliferation of murine pancreatic cancer cells in vivo (Fig. 5d and e). In agreement with the observation that Pd-l1 protein was detectable in Panc 02 cells (Fig. 5a), the anti-PD-1-antibody treatment impeded tumor growth and prolonged the survival time of tumor-bearing mice (Fig. 5d and e). Furthermore, the knockdown of Hat1 significantly increased tumor infiltration of immune effectors including CD45^+^CD8^+^ T cells and CD45^+^CD4^+^ T cells, but decreased the infiltration of CD11b^+^Gr1^+^ myeloid cells in tumors (Fig. 5f). In agreement with the effect on tumor regression, treatment with the anti-PD-1-antibody after the knockdown of Hat1 led to a further decrease in tumor growth and an increase in CD45^+^CD8^+^ and CD45^+^CD4^+^ T cell infiltration, but a greater decrease in CD11b^+^Gr1^+^ myeloid cells in tumors (Fig. 5f). Therefore, our findings indicate that the knockdown of HAT1 improves the efficacy of immune checkpoint blockade by decreasing the PD-L1 expression and in vivo.

### HAT1 increases PD-L1 expression through BRD4 in pancreatic cancer cells

**Fig. 6:**
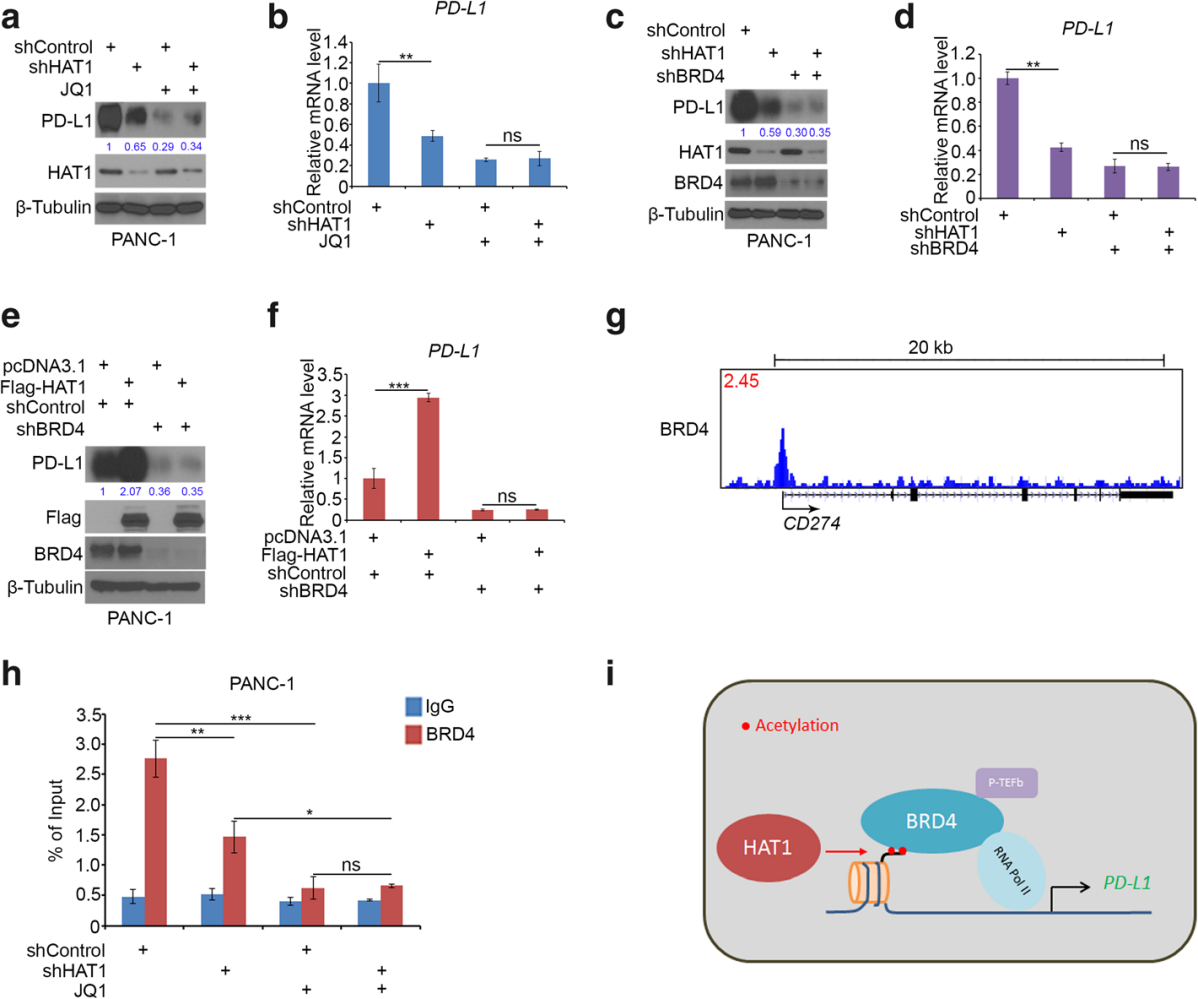
HAT1 increases PD-L1 expression through BRD4 in pancreatic cancer cells. **a** and **b**, PANC-1 cells were infected with lentivirus vectors expressing control or HAT1-specific shRNAs. Forty-eight hours postinfection, the cells were treated with or without JQ1 (3 μM) for another 24 h. Cells were harvested for Western blotting (**a**) and RT-qPCR analysis (**b**). The data shown are the mean values ± SD from three replicates. ns, not significant; **, *P* < 0.01. **c** and **d**, PANC-1 cells were infected with the indicated constructs. After 48 h, the cells were harvested for Western blotting (**c**) and RT-qPCR analysis (**d**). The data shown are the mean values ± SD from three replicates. ns, not significant; **, *P* < 0.01. **e** and **f**, PANC-1 cells were infected with lentivirus vectors expressing control or BRD4-specific shRNAs. Forty-eight hours postinfection, the cells were transfected with pcDNA 3.1 or Flag-HAT1. After 24 h, the cells were harvested for Western blotting (**e**) and RT-qPCR analysis (**f**). The data shown are the mean values ± SD from three replicates. ns, not significant; ***, *P* < 0.01. **g**, UCSC Genome Browser screenshots of the BRD4 ChIP-seq profiles at the PD-L1 gene locus in C4–2 cells reported previously [27]. **h**, PANC-1 cells were infected with lentivirus vectors expressing control or HAT1-specific shRNAs. Forty-eight hours postinfection, the cells were treated with or without JQ1 (3 μM) for another 24 h. The cells were harvested for ChIP-qPCR analysis (**h**). The data shown are the mean values ± SD from three replicates. ns, not significant; *, *P* < 0.05; **, *P* < 0.01;***, *P* < 0.001. **i**, A hypothetical model depicting the catalysis of histone H4 acetylation by HAT1 and the BRD4 complex binding to the acetylated H4 to initiate the transcription of PD-L1

Although HAT1 transcriptionally increased PD-L1 expression, but the underlying mechanism is not fully understood. It has been documented that various transcriptional factors, such as STAT3, MYC, p65 and BRD4, could directly bind to the promoter of PD-L1 and regulate the transcription of PD-L1 in cancer cells. Moreover, HAT1 catalyzes the acetylation of H4K5 and H4K12, which is essential for BRD4 binding to the histone H4 and initiating the transcription. Herein, we hypothesized that BRD4 was the key protein that mediates the HAT1-induced PD-L1 expression. To test this hypothesis, we knocked down HAT1 in PANC-1 cells and treated them with or without the BRD4 inhibitors JQ1 (Fig. 6a and b). We found that the knockdown of HAT1 repressed PD-L1 expression and that these effects were diminished by JQ1 treatment (Fig. 6a and b). Similarly, the knockdown of the HAT1 induced PD-L1 downregulation was not significant after the knockdown of BRD4 in PANC-1 cells (Fig. 6c and d). Conversely, the ectopic expression of HAT1 significantly increased the PD-L1 expression, but these effects were not apparent after the knockdown of BRD4 in PANC-1 cells (Fig. 6e and f). Notably, just as reported by other groups, we checked the existing BRD4 ChIP-seq data [27] and noticed that there is a BRD4 binding peak in the promoter of the *PD-L1* gene (Fig. 6g). Thus, we confirmed the binding of BRD4 by ChIP-qPCR in PANC-1 cells (Fig. 6h). Consistently, JQ1 prevented BRD4 binding to the promoter of PD-L1 and abolished the effect of the HAT1 knockdown-induced reduction in BRD4 binding to the PD-L1 promoter (Fig. 6h). Collectively, these data suggest that HAT1 catalyzes the histone H4 acetylation and that the BRD4 complex binds to the acetylated H4 to initiate the transcription of PD-L1 (Fig. 6i).

## Discussion

Histone acetyltransferases (HATs) and histone deacetylases (HDACs) influence DNA transcription through the balance between histone acetylation and deacetylation [28]. HAT1 acetylates newly synthesized histone H4 but not nucleosomal histones and regulates the genes involved in cell differentiation, proliferation, cell metabolism and apoptosis [14–16]. HAT1 plays a critical role in the tumorigenesis of digestive system cancer. It has been well documented that HAT1 is an important determinant in the regulation of the proliferation of esophageal and liver cancer cells in vivo and in vitro [14, 16]. Moreover, HAT1 is overexpressed in multiple types of cancer and associated with poor prognosis [29]. In this study, we demonstrate that HAT1 is upregulated and highly correlated with poor prognosis in pancreatic cancer specimens. The aberrant expression of HAT1 participates in promoting tumor cell growth in pancreatic cancer.

The nonimmunogenic characteristics of pancreatic cancer are responsible for the failure of immunotherapy. Only a small portion of pancreatic cancer patient specimens were positive for PD-L1, representing the best candidates for PD-L1 blockade therapy. However, monotherapy with PD-L1 blockade has no effect on the survival time of pancreatic cancer patients. Given that the expression level of PD-L1 plays a key role in determining the efficacy of anti-PD-L1 therapy, understanding the regulatory mechanism of PD-L1 in cancer cells sheds new light on the exploration of novel therapy strategies for cancer treatment. Recent studies showed that various transcriptional factors, including BRD4 [30], MYC [31], p65 [32] and STAT3 [33], could directly bind to the PD-L1 promoter and initiate PD-L1 transcription. Moreover, RAS signaling is involved in regulating the mRNA stabilization of PD-L1 in cancer cells [34]. Furthermore, beyond the mRNA level regulation, it has been documented that the E3 ligase, SPOP [12], β-TrCP [35], and the deubiquitinase CSN5 [36] participate in modulating the stability of PD-L1 through the proteasome pathway. In addition, the transmembrane proteins, CMTM4 and CMTM6, stabilize the PD-L1 protein via the lysosome pathway [37, 38]. In our study, we demonstrate that HAT1 is a novel regulator of PD-L1 at the transcriptional level and that BRD4 might be an important mediator of this process.

Our data indicated that the knockdown of HAT1 blocked pancreatic cancer tumor growth (Fig. 2), but the overexpression of HAT1 promoted the tumor growth in vivo (Additional file 1: Figure S1). Our results further showed that HAT1 regulated PD-L1 expression in pancreatic cancer (Fig. 3), and PD-L1 has been reported to promote tumor cell growth not only via immune effects but also through tumor cell-intrinsic signals, including the regulation of autophagy and the mTOR pathway [39]. Therefore, HAT1 might regulate cancer cell proliferation via PD-L1. It has been well documented that HAT1 could regulate the function of BRD4 or increase the acetylation level of histones to influence the expression of a number of genes involved in apoptosis and glucose metabolism [15, 16, 25], which was critical for the cell viability and not involved in the PD-L1 effect. Therefore, PD-L1 is partially responsible for promoting the pancreatic cancer cell growth induced by HAT1 in vivo, and this is confirmed by our results in Additional file 1: Figure S2.

Our data indicated that HAT1 increased the PD-L1 expression in vivo and in vitro, which identifies HAT1 as a previously unrecognized master regulator of this critical immune checkpoint. However, there is no small molecular inhibitor to specifically target HAT1. Recent studies have indicated that ascorbate represses the HAT1 expression via the TET-mediated DNA hydroxymethylation pathway [25]. Our findings suggest that ascorbate could suppress PD-L1 expression by influencing the HAT1 level in pancreatic cancer cells. Although ascorbate is not a the specific inhibitor of HAT1, it may regulate PD-L1 expression through other pathways. These data also suggest that ascorbate might be a potential new avenue to overcome immune evasion by tumor cells.

## Conclusions

In summary, we proposed a new understanding of the specific role of HAT1 in pancreatic cancer. We showed that HAT1 is overexpressed in pancreatic cancer specimens and highly correlated with poor prognosis in pancreatic cancer. Furthermore, our results suggested that HAT1 promoted cell proliferation in pancreatic cancer cells. In particular, we demonstrated that HAT1 functioned as an important regulator in cancer immunity via transcriptionally upregulating the PD-L1 level in tumor cells. The recognition of HAT1 in the regulation of PD-L1 expression suggests that HAT1 might be employed as a new diagnostic and prognostic marker and as a predictive marker for pancreatic cancer therapy, especially in immune checkpoint blockade therapy. Targeting HAT1 highlights a novel therapeutic target to overcome immune evasion by tumor cells.

## Additional file

Additional file 1: Overexpressed histone acetyltransferase 1 regulates cancer immunity by increasing programmed death-ligand 1 expression in pancreatic cancer. (PDF 544 kb)

## Abbreviations

ChIP: Chromatin immunoprecipitation
HAT1: Histone acetyltransferase 1
IHC: immunohistochemistry
MTS: 3-(4,5-dimethylthiazol-2-yl)-5-(3-carboxymethoxyphenyl)-2-(4-sulfophenyl)-2H-tetrazolium, inner salt; TMA: tissue microarray
PDAC: Pancreatic ductal adenocarcinoma
PD-L1: Programmed death-ligand 1
