# Supplementary material for: Overexpressed histone acetyltransferase 1 regulates cancer immunity by increasing programmed death-ligand 1 expression in pancreatic cancer
Source: J Exp Clin Cancer Res. 2019 Feb 1;38:47. doi: 10.1186/s13046-019-1044-z (PMC6359760; doi:10.1186/s13046-019-1044-z)
Supplement: Supplementary file 1 — Overexpressed histone acetyltransferase 1 regulates cancer immunity by increasing programmed death-ligand 1 expression in pancreatic cancer. (PDF 544 kb) [file 13046_2019_1044_MOESM1_ESM.pdf]

Overexpressed histone acetyltransferase 1 regulates cancer immunity by increasing programmed death-ligand 1 expression in pancreatic cancer

Ping Fan, Jingyuan Zhao, Zibo Meng, Heyu Wu, Bo Wang, Heshui Wu and Xin Jin

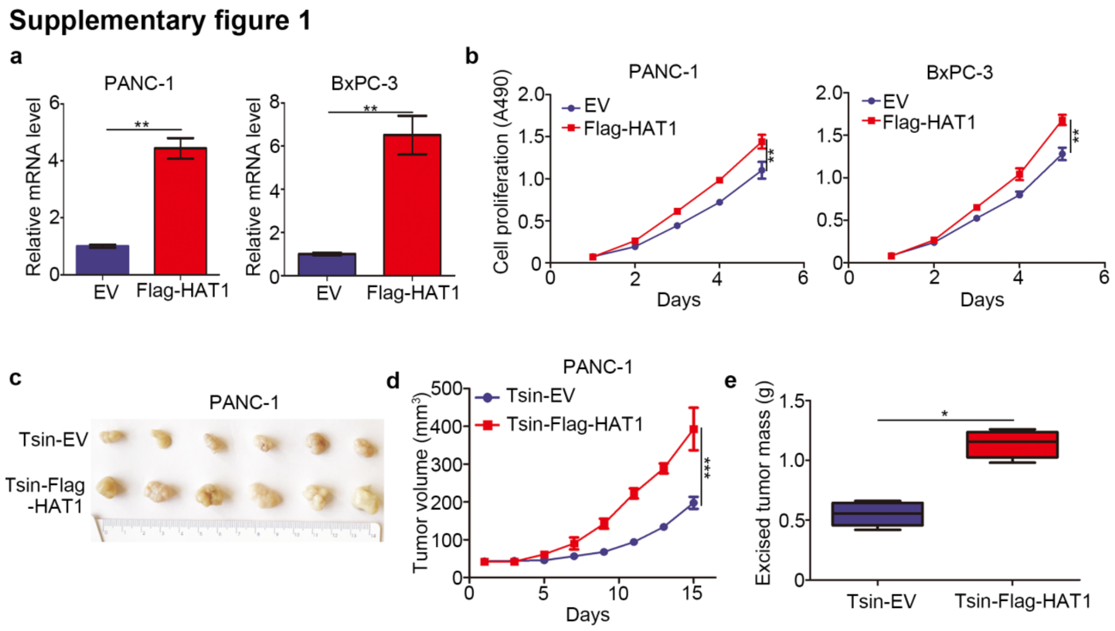

Supplementary figure 1. Overexpressed HAT1 promotes pancreatic cancer growth in vivo and in vitro. a and b, PANC-1 and BxPC-3 cells were transfected with indicated constructs. Twenty-four hours postinfection, the cells were harvested for RT-qPCR analysis (a), MTS assay (b). The data shown are the mean values ± SD from three replicates. **, $P < 0.01$. **c-e**, PANC-1 cells were infected with lentiviral vectors as indicated. Seventy-two hours after selection with puromycin, the cells were injected subcutaneously into the right flank of nude mice and tumor growth was measured for 15 days. Tumors in each group at day 15 were harvested, photographed,

and are shown in c. Data in d and e are shown as means ± SD (error bar) from five replicates. *, $P < 0.05$; ***, $P < 0.001$.

Supplementary figure 2

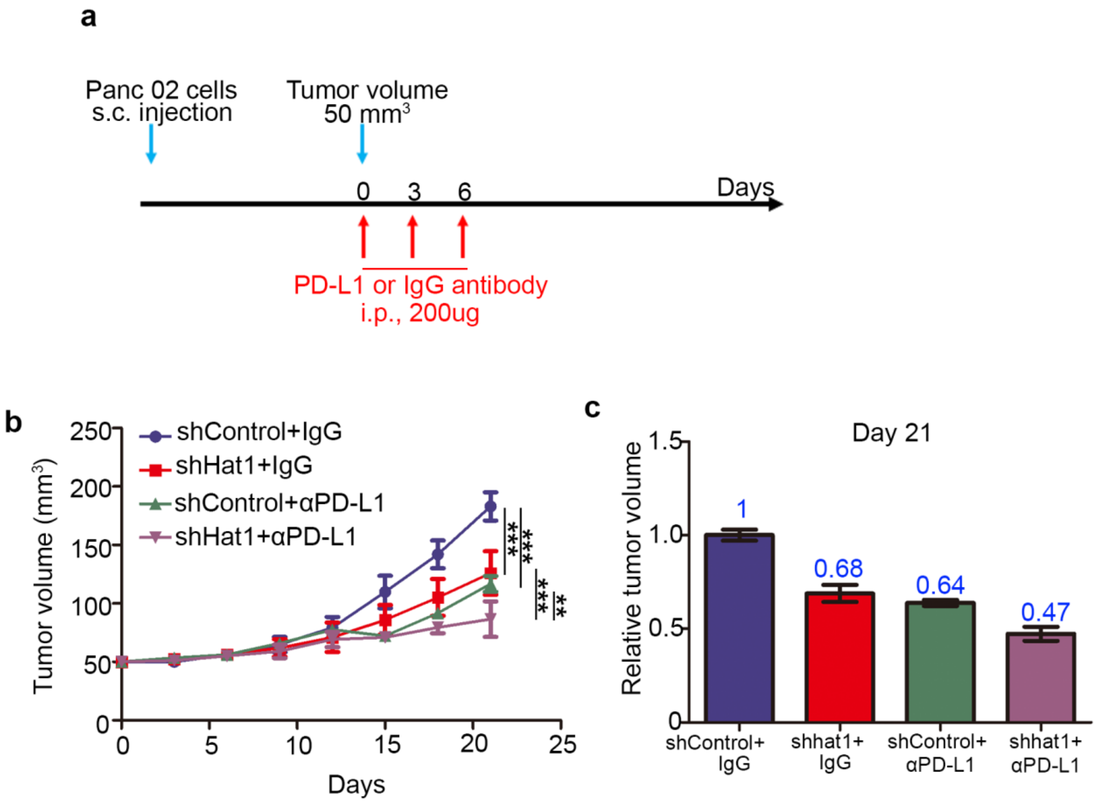

Supplementary figure 2. PD-L1 is partially responsible for promoting the pancreatic cancer cell growth induced by HAT1 in vivo. a, Schematic diagram depicting the treatment plan for mice bearing subcutaneous Panc 02 tumors**. b and c,** Panc 02 cells were infected with lentivirus vectors expressing control or Hat1-specific shRNAs. Seventy-two hours after selection with puromycin, 5 x 10^6 cells were injected subcutaneously into C57BL/6 mice. Mice (n=5/group) were treated with anti-PD-L1 (200 μg) or non-specific IgG for 21 days. The growth curves of tumors

with the different treatments are shown in (b). The tumor growth volume in day 21 was shown in (c). All data are shown as the mean values ± SD. ns, not significant, * $P < 0.05$, ** $P < 0.01$, *** $P < 0.001$.

Supplementary Table S1: Sequences of RT-qPCR primers

**Species**	**Gene**	**Forward (5'-3')**	**Reverse (5'-3')**
Human	*β-actin*	GACCTCTATGCCAACACAGT	AGTACTTGCGCTCAGGAGGA
Human	*PD-L1*	GGTGCCGACTACAAGCGAAT	AGCCCTCAGCCTGACATGTC
Human	*HAT1*	GGATGGAGCTACGCTCTTTG	GGATGGATCTTCCGCTGTAA
Mouse	*Gapdh*	AGGTTGTCTCCTGCGACTTCA	GGGTGGTCCAGGGTTTCTTACT
Mouse	*Pd-l1*	AATGCTGCCCTTCAGATCAC	ATAACCCTCGGCCTGACATA

Supplementary Table S2. Sequences of ChIP-qPCR primers

**Species, ChIP target**	**Gene**	**Forward (5'-3')**	**Reverse (5'-3')**
Human, BRD4	*PD-L1*	AAGCCATATGGGTCTGCTC	TTATCAGAAAGGCGTCCCCC

Supplementary Table S3: Sequences of gene-specific shRNAs

shHAT1-1	5′- CCGGGCTACATGACAGTCTATAATTCTCGAGAATTATAGACTGTCATGTAGCTTTTTG-3′
shHAT1-2	5′- CCGGCCGTGTTGAATATGCATCTAACTCGAGTTAGATGCATATTCAACACGGTTTTTG-3′
shHAT1-3	5′-CCGGGCAAGGATTCAATGAAGATATCTCGAGATATCTTCATTGAATCCTTGCTTTTTG-3′
shHat1-1	5′-CCGGCCGTGTTGAATATTCATCTAACTCGAGTTAGATGAATATTCAACACGGTTTTTG-3′
shHat1-2	5′-CCGGCCAGAAGCTATTTGAAACTAACTCGAGTTAGTTTCAAATAGCTTCTGGTTTTTG-3′
shHat1-3	5′-CCGGGCAAGGATTCAGTGAAGATATCTCGAGATATCTTCACTGAATCCTTGCTTTTTG-3′
shBRD4-1	5′ -CCGGCCTGGAGATGACATAGTCTTACTCGAGTAAGACTATGTCATCTCCAGGTTTTTG-3′
shBRD4-2	5′ -CCGGCAGTGACAGTTCGACTGATGACTCGAGTCATCAGTCGAACTGTCACTGTTTTTG-3′
